# A Low-Power Analog Integrated Implementation of the Support Vector Machine Algorithm with On-Chip Learning Tested on a Bearing Fault Application

**DOI:** 10.3390/s23083978

**Published:** 2023-04-14

**Authors:** Vassilis Alimisis, Georgios Gennis, Marios Gourdouparis, Christos Dimas, Paul P. Sotiriadis

**Affiliations:** Department of Electrical and Computer Engineering, National Technical University of Athens, 15780 Athens, Greece

**Keywords:** support vector machine, bulk-controlled circuits, low-power design, bearing fault application, analog-hardware implementation

## Abstract

A novel analog integrated implementation of a hardware-friendly support vector machine algorithm that can be a part of a classification system is presented in this work. The utilized architecture is capable of on-chip learning, making the overall circuit completely autonomous at the cost of power and area efficiency. Nonetheless, using subthreshold region techniques and a low power supply voltage (at only 0.6 V), the overall power consumption is 72 μW. The classifier consists of two main components, the learning and the classification blocks, both of which are based on the mathematical equations of the hardware-friendly algorithm. Based on a real-world dataset, the proposed classifier achieves only 1.4% less average accuracy than a software-based implementation of the same model. Both design procedure and all post-layout simulations are conducted in the Cadence IC Suite, in a TSMC 90 nm CMOS process.

## 1. Introduction

There is a growing trend towards using more sophisticated design concepts for the development of new sensor systems, especially for so-called smart sensor systems that integrate sensing elements with signal processing, conversion, and output units [1,2]. These modern smart sensor systems employ an increasing number of sensors to sense a range of physical variables, thanks to continuous advancements in technology that offer promising solutions in miniaturization and power-efficiency [3]. Integrated circuit (IC) technologies have resulted in complex but power- and area-efficient devices that address the challenges of smart sensor systems. This is particularly true for analog ICs, which can achieve high-performance computations based on the physical laws of MOS or BJT transistors [4,5]. In analog computing, various mathematical equations and models can be efficiently approximated using analog ICs. These models are used in machine learning (ML) applications that, in the case of real-time interactions, can benefit from the efficiency of ICs. However, digital implementations usually require power-hungry analog-to-digital conversions compared to analog implementations [6].

**Figure 1:**
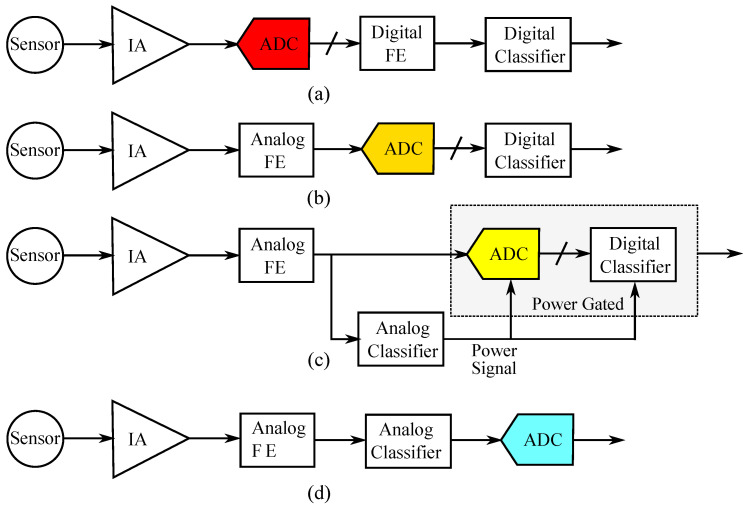
Different architectures for a classification system. (**a**) All digital inference; only the sensor related circuitry is analog. (**b**) The analog feature extractor replaces the digital one. (**c**) The analog front-end is used as a wake-up circuit in order to power up/down the digital back-end. (**d**) A pure analog approach. From (**a**–**d**), the power requirements of the ADC are reduced.

To extract useful information, a typical hardware-friendly ML classification system contains a sensor, an instrumentation amplifier (IA) or an analog front-end for signal processing, a feature extractor (FE) block, and a classifier [7,8]. In the traditional approach, only the sensor-related circuitry is analog, and a power-costly ADC is used to convert raw analog data to digital for further processing [9]. In this configuration, the (possibly strong) correlation and redundancy in the high-rate raw data are not useful to the digital feature extractor, as shown in Figure 1a. Therefore, to minimize the ADC’s conversion rate and reduce power consumption, the feature extraction part can be shifted to the analog domain, as presented in Figure 1b [5,10,11]. This way, only a small amount of uncorrelated analog data is converted to digital. The next step towards pure analog computing is the use of simple analog-based ML models (which cannot achieve high accuracies) as wake-up circuits, as shown in Figure 1c [8]. In this case, the analog ML models are probably not accurate enough to operate autonomously, but their inclusion benefits the overall system in terms of power consumption by minimizing the use of the digital classifier. In other words, an analog classifier decides when the ADC and the digital classifier are turned on. Therefore, the power-hungry digital components operate for only a fraction of the overall time, reducing the system’s time-average power consumption. With constant advancements in analog ML circuits, the digital back-end processing is diminished [12]. It is important to note that the key characteristic of the pure analog approach, presented in Figure 1d, is its very low power consumption, which for certain battery-dependent applications is critical.

In the literature, a variety of ML algorithms and models (classifiers) have been implemented in analog hardware. This includes radial basis function (RBF) neural networks (NN) [13] or Gaussian RBF networks (GRBFN) [14], Gaussian mixture model (GMM) [15], Bayesian [16], K-means-based [17] classifiers, voting classifier [18], support vector regression [19], NN classifiers [20,21], deep machine learning engine [22], artificial NN implemented Gaussian kernel functions [23], and anomaly detection circuits [24]. It is important to note that, although these classifiers may seem different, they can all be similarly employed in various classification tasks, regardless of the implemented ML model. It is also worth noting that the training procedure for these classifiers is not implemented in silicon and requires external assistance. In this work, a fully autonomous classifier is proposed, and the necessary circuitry for training the support vector machine (SVM) algorithm is also included in the design.

A highly researched topic in the literature is the hardware implementation of SVMs to be used as classifiers. In digital implementations, this involves FPGA-based architectures [25,26,27,28,29]. There have also been several mixed signal [30] and analog [31,32,33,34] architectures for hardware implementation of SVMs.

The work presented in [31] utilizes an array of analog translinear circuits with floating gate transistors operating in the subthreshold region to implement a quadratic kernel SVM classifier. The low power computation provided by translinear and subthreshold techniques is combined with analog non-volatile memory storage due to the existence of floating gate transistors. This specific implementation achieves very low power consumption, regardless of the very large-scale setup. It performs multi-class SVM for 24 classes, with input vectors of 14 dimensions and as many as 720 support vectors. However, the learning procedure is not performed on-chip, as a PC-in-loop technique is used instead, where a computer is connected to the system that performs the update of the learning parameters of SVM in software. These parameters are then downloaded to the analog floating gate array. In contrast, the circuit architectures presented in [32,33,34] perform on-chip learning and classification based on the SVM.

In reference [32], a fully analog implementation of the SVM using floating gate transistors operating in the subthreshold region is presented. To implement the learning procedure, projection neural networks adapted for SVM are proposed, and the constrained quadratic problem is solved by a set of ordinary differential equations. However, this fully analog approach has only been realized through MATLAB and Spice simulations, without an actual analog VLSI design taking place. This is reasonable because the analog circuit design and tape-out of such an architecture would be complicated due to the presence of floating gate transistors.

In reference [33], a row-parallel architecture is presented that uses transistors operating in the subthreshold region. It employs a hardware-friendly version of the SVM algorithm that is also used in this work. The proposed implementation includes the learning circuit and is area-efficient while achieving low power consumption. However, the proof of concept chip fabricated as part of this work can only classify input vectors of 2 dimensions. Additionally, to implement the training mode of the SVM, an ADC and a digital block in a feedback loop configuration realizing a binary search algorithm are necessary.

In reference [34], a fully analog and parallel architecture is presented. The basic circuit components of this architecture enable an area-efficient implementation of analog kernels, as well as a more robust design compared to other works, suitable for implementing high-dimensional kernels accommodating inputs of up to 64 dimensions each. This architecture also makes use of the hardware-friendly SVM algorithm but realizes it with fully analog circuitry [33]. The analog circuits are self-converging, determining the proper Lagrange multiplier values for SVM learning without the presence of an external digital clock. For the realization of multivariate RBF kernels, this architecture uses circuits with transistors operating in the saturation region. While this design choice increases the speed of operation and the robustness of the architecture against process variations, it leads to higher power consumption compared to implementations exclusively using transistors operating in the subthreshold region.

Motivated by the need for low-power smart sensors [35,36] we combine subthreshold-based analog computing techniques with ML ones [37]. To this end, in this work, an analog, integrated, low-voltage (0.6 V), low-power (72 μW) SVM model with on-chip learning is introduced and tested on a bearing fault management classification problem. It is realized based on the hardware-friendly mathematical model proposed in in [33], using a variety of sub-circuits. Specifically, ultra-low power Gaussian function circuits [38], multiplier circuits [39], switch cells [39], adjuster circuits [39], and an argmax operator circuit [40] are employed as building blocks. The classifier is trained and tested on a real-world bearing fault management dataset [41]. Post-layout simulation results are conducted on a TSMC 90 nm CMOS process using the Cadence IC suite and compared with a software-based implementation. Additionally, Monte Carlo analysis confirms the proper sensitivity of the implemented architecture.

The current implementation is designed to operate in the subthreshold region, with the aim of reducing power consumption in comparison to state-of-the-art publications [30] and analog ones [31,32,33,34]. Specifically, it employs a power supply of only 0.6 V and has a low bias current. Furthermore, by controlling the bulk of the MOS transistors, we are able to manipulate parameters that were not adjustable in prior implementations (in the case of the Gaussian function circuit). Our implementations based on mathematical approaches leverage the subthreshold region and bulk-controlled techniques, thereby eliminating the need for additional analog (exponentiator, absoluter, translinear loops, etc.) or digital or conversion (ADC, digital memories, etc.) blocks.

The remainder of this paper is organized as follows. Section 2 refers to the hardware-friendly mathematical model of this work. More specifically, the SVM learning and classification rules are explained. The proposed high-level architecture of the analog integrated SVM implementation is presented in Section 3. The main basic building block for the learning and the classification blocks is thoroughly analyzed in Section 4. The proper operation of the implemented classifier is confirmed via a real-world bearing fault management dataset in Section 5. A performance summary regarding analog SVM classifiers is provided in Section 6. Concluding remarks are given in Section 7.

## 2. Hardware-Friendly SVM

An SVM-based classifier is a classic binary classification algorithm in which the Lagrange multipliers’ values are determined by solving the constrained quadratic programming problem. The gradient-descent algorithm that is usually used for solving this problem is:

(1) $$a_{i}\overset{}{\leftarrow }a_{i}-\frac{\partial W(a,b)}{\partial a_{i}}n_{i},$$

where $n_{i}$ is the learning rate and *a*,*b* are the bias values. However, this SVM learning rule can be modified to be more compatible with analog hardware. In this work, a hardware-friendly version of the SVM learning rule, which was first introduced in [33] and also used in [34], is adopted.

For choosing the learning rate equal to

(2) $$n_{i}=\frac{1}{K(x_{i},x_{i})},$$

and in the case of *K* being a self-normalized kernel like the Gaussian kernel ($K(x_{i},x_{i})=1$), the hardware-friendly SVM update rule is defined as follows:

(3) $$a_{i}\overset{}{\leftarrow }min\left(C,max\left(0,1-y_{i}\sum_{i\neq m}y_{m}a_{m}K\left(x_{i},x_{m}\right)\right)\right).$$

In this update rule, the bias value *b* is set to 0. The characteristics of the Gaussian kernel, which maps the input vectors to a space of infinite dimensions, makes the omission of a single bias value *b* possible, as its effect on the total result can be considered negligible.

The derived SVM update rule of the last equation is more suitable for hardware implementation, thanks to the specific properties it demonstrates. First, there is no need for extra memory to store previous $a_{i}$ values, as they do not appear in the right-hand side of the update rule. Furthermore, the form of the update rule resembles that of the classification rule (4), meaning that common hardware blocks could be used for both tasks. This would simplify the system architecture and make it more compact and area-efficient. The classification rule is given by

(4) $$f\left(x\right)=sign[\sum_{i=1}^{N}a_{i}y_{i}K\left(x,x_{i}\right)+b],$$

for input test vector *x* and a training set $[x_{i}$,$y_{i}{]}_{i=1}^{N}$.

## 3. Proposed High-Level Architecture

**Figure 2:**
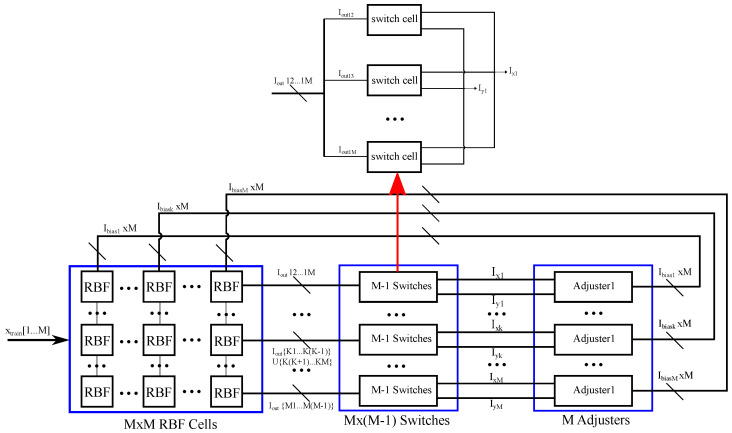
An illustration of the learning block. The RBF cells receive the learning samples and output the multivariate RBF kernel. The input labels are imported to the training circuit via the following switches. The adjusters implement the min and max operators.

In this section, the proposed classifier’s high level architecture and its two main blocks is discussed. The first one, shown in Figure 2, is related to the classifier’s learning and contains the hardware-friendly, rule-based ML methods.

**Figure 3:**
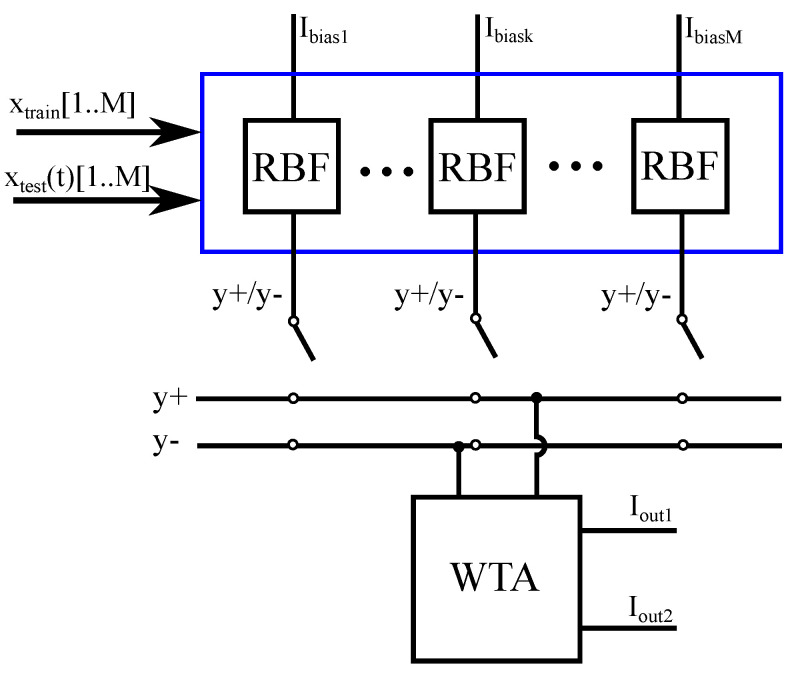
The prediction block of the classifier (classification block). The RBF cells receive the input and produce the appropriate RBF functions based on the trained parameters. These RBF functions represent the support vectors. The sign of the support vectors is imported to the classification block by the switches. The WTA is used to compare the positive and the negative values.

From a system-level perspective, the learning block is designed to realize the update rule of the hardware-friendly SVM. In practice, there is a need for circuits that realize the Gaussian kernels, multiply with a specific value $a_{i}$, incorporate labels, and perform the appropriate iterations of the learning rule. The second block, depicted in Figure 3, aims to implement the SVM’s decision rule in (4) in hardware. It shares certain common building blocks with the learning block due to the resemblance of the two realized mathematical expressions. However, the classification block also contains circuits that determine the *sign* of a summed expression or that perform the argmax operator. In both the learning and classification blocks, the Lagrange multipliers’ and kernel function’s values are realized with transistor currents, while the labels $y_{i}=+-1$ correspond to the positive and negative supply voltages, respectively. The learning block receives *M* vectors of *N* dimensions as inputs (learning samples) along with *M* corresponding labels and produces *M* output currents, which represent the Lagrange multiplier values. These current values are inserted as parameters to the classification block together with *M* learning samples (support vectors) and their *M* labels. Periodically, the classification block receives a new input vector of *N* dimensions (test sample) and produces a set of output currents with binary values that encode the classifier’s decision in a one-hot-vector format.

### 3.1. Learning Block

The learning block is composed of an array of $M^{2}$ RBF cells, where *M* is the number of samples involved in the learning procedure. The learning samples, which are the inputs of the system, are received by the RBF cells. In practice, each RBF cell implements a multivariate RBF kernel of *N* dimensions. The M(M−1) switches provide the appropriate input labels to the learning block. The output of every $X_{i,j}$ RBF cell, for i≠j, from the matrix $X_{M\cdot M}$ of the RBF cells, is inserted into a single switch cell. Here, the switch cell implements an operation between the label values of the corresponding row and column. Depending on the result of the operation, the output current of each RBF cell is driven through one of the two outputs of the switch cell ($I_{xi}$ and $I_{yi}$). For every row of the RBF cells’ $X_{M\cdot M}$ matrix, the output currents that have the same operation results are summed together. Each of these currents corresponds to a specific input learning sample of the block. Then, each branch of summed currents is connected to the appropriate input of an adjuster circuit.

In the aforementioned case, there are *M* adjuster circuits that essentially implement the non-linear min–max operations of the hardware-friendly update rule. The summed output currents for the row *j* of the matrix $X_{M\cdot M}$ that are produced by the RBF cells are received by an adjuster circuit whose output current is fed back to the bias current for the RBF cells of the column *j*. Thus, a feedback loop configuration is formed, and the learning circuitry self-converges without the use of an external clock. The learning process is completed in a fully parallel and autonomous fashion, determining the correct values for the adjusters’ output currents, which represent the learning parameters of the SVM algorithm.

### 3.2. Classification Block

The classification block consists of *M* RBF cells, *M* switches, and a winner-take-all (WTA) circuit (argmax operator circuit). The test samples (vectors of *N* dimensions) are synchronously (based on an external clock) fed to the classification block. During every clock cycle, each of the *M* RBF cells computes the RBF kernel function of the cycle’s test vector based on the learning samples that were used in the training procedure. In practice, the RBF cells of the classification block are biased with copies of the adjusters’ output currents of the learning block.

In order to determine the classifier’s prediction, the *sign* of the sum in Equation (4) of the SVM’s decision rule has to be calculated. To do so, instead of adding all of the currents together and inspecting whether the sum is positive or negative, we add the positive and the negative currents separately. This can be easily achieved since the positive (or negative) currents are ones that correspond to an input learning sample with a positive (or negative) label. This separation is implemented with switches, and the comparison between the negative and positive values is achieved through a current-mode circuit called WTA circuit. The WTA’s output encodes the classifier’s prediction into a one-hot-vector format ($\left[I_{out1},I_{out2}\right]$). A WTA circuit is used instead of a comparator due to the fact that information processing in the system is performed mainly in current-mode.

## 4. Circuit Implementation

The main building circuits for both the learning and the classification blocks are thoroughly analyzed in this section. Based on Section 3, the learning block requires three main cells: an RBF, a switch, and an adjuster (min–max operator) cell. On the other hand, for the classification block, two main building blocks are needed: an RBF cell and an argmax operator circuit. The whole architecture aims at utilizing ultra-low-power circuits as building blocks for implementing the main cells and hence all transistors of the architecture operate in the subthreshold region. To enhance the classifier’s applicability in battery-dependent cases, the power supply rails are set to $V_{DD}=-V_{SS}=0.3\;V$. The proposed architecture was tested on a real-world dataset [41], for both learning and classification, using 8 learning samples of 13 dimensions.

### 4.1. Gaussian Function Circuit

Each RBF cell in the proposed system architecture is composed of a multidimensional Gaussian function circuit (specifically bump circuits) and an analog multiplier. Gaussian function circuits are analog circuits that produce a univariate Gaussian function as their output [15,38].

**Figure 4:**
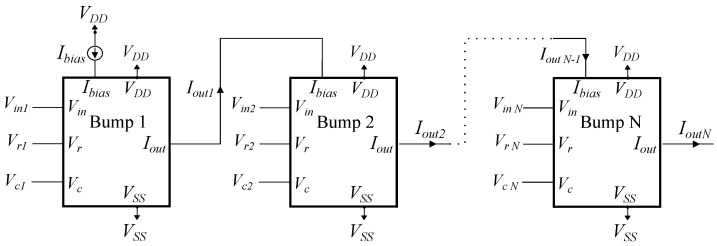
By connecting *N* simple bump circuits sequentially, the output of the last one is equivalent to an *N*–D Gaussian function. Each bump circuit’s parameters ($V_{r}$, $V_{c}$, $I_{bias}$) are tuned independently.

Bump circuits are preferred for implementing multivariate Gaussian functions because two or more bump circuits can be connected in a cascaded format, and the output of the last bump is equal to their multiplication [42]. This approach works well for a Gaussian function with a diagonal covariance matrix, since the multivariate function can be calculated as the multiplication of the individual univariate ones. An example of a multidimensional Gaussian function circuit is shown in Figure 4. In this configuration, only the first bump circuit is biased with a current *Ibias*, and the last bump circuit’s output is used as input current for the analog multiplier.

The multiplier adjusts the height of the Gaussian function, and its output current is the output of the entire RBF cell.

**Figure 5:**
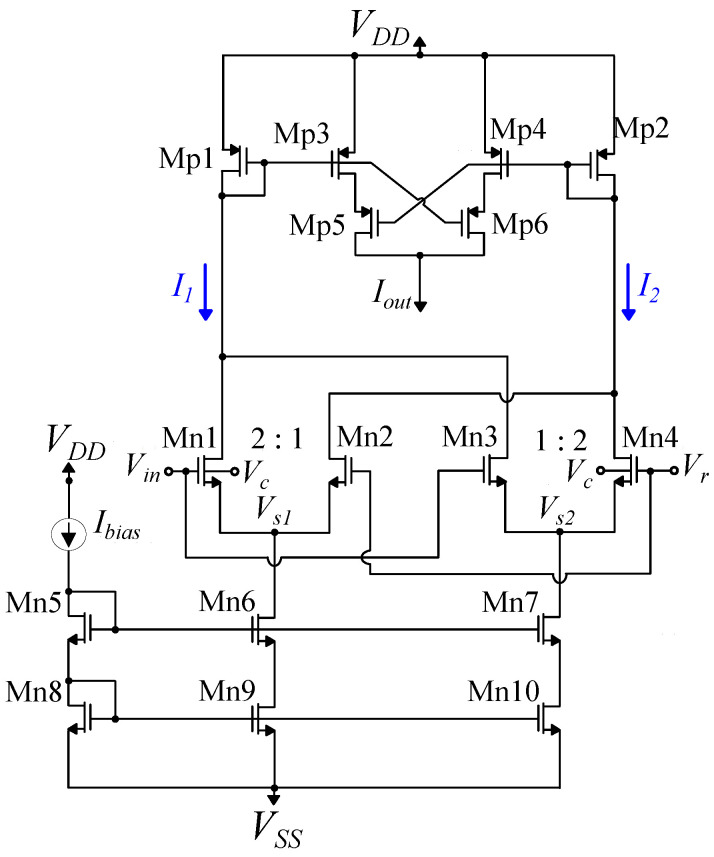
The utilized Gaussian function circuit is presented. The output current $I_{out}$ resembles a Gaussian function controlled by the input voltage $V_{in}$. The parameter voltages $V_{r}$, $V_{c}$, and the bias current $I_{bias}$ control the Gaussian function’s mean value, variance, and peak value, respectively.

The original bump circuit was proposed by Delbruck [43] and, since then, there have been numerous implementations following different design approaches for realizing a Gaussian function in analog hardware [38]. The primary challenges in designing Gaussian function circuits are usually low power consumption, accurate approximation of the Gaussian function, as well as independent and electronic tunability of the Gaussian function’s characteristics (height, mean value, and variance). The Gaussian function circuit used in the proposed system, depicted in Figure 5, was firstly proposed in [15,44]. It consists of two main building blocks, a differential difference pair ($M_{n1}$–$M_{n4}$) and a symmetric current correlator ($M_{p1}$–$M_{p6}$), along with transistors $M_{n5}$–$M_{n10}$ that form the cascode current mirrors used for biasing. Each bump circuit receives a unique input voltage *Vin* and two parameter voltages Vr and Vc. The output current of the current correlator is a Gaussian function of *Vin*, with parameters $I_{bias}$, Vr, and Vc adjusting the height, the mean value, and the variance of the Gaussian function output, respectively [15,44]. Thus, the proposed circuit exhibits electronic tunability of all the Gaussian function’s characteristics. All the transistors’ dimensions in the circuit are summarized in Table 1.

The proposed Gaussian function circuit possesses several essential characteristics that make it a fundamental building block of the proposed system architecture [15,44]. Firstly, the use of cascode current mirrors, instead of simple ones, provides precise biasing for the differential difference pair, resulting in accurate current mirroring even for very small currents, as low as 1 nA. Moreover, compared to a simple current correlator, the symmetric current correlator used in the circuit improves the symmetry of the Gaussian function output curve. These modifications result in a more robust circuit architecture suitable for high-dimensional RBF kernel applications, although they require extra transistors, which increase the circuit area. For a detailed explanation of the circuit’s operation, as well as mathematical analysis and simulation results, refer to [15,44].

A limitation of this design, however, is that when the number of bump cells in such a cascaded implementation is increased in order to accommodate high-dimensional data, the current scaling caused by the $I_{bias}$ is not entirely linear. This loss of linearity can be attributed to small inaccuracies of analog circuits, which may be negligible for low dimensional inputs; however, as more bumps are connected in series, these inaccuracies accumulate and affect the output current considerably. In the SVM case particularly, the bias current of each cascaded bump circuit is the parameter that gets updated during the learning procedure, so linear scaling of the RBF’s output’s current is of paramount importance.

### 4.2. Multiplier Circuit

**Figure 6:**
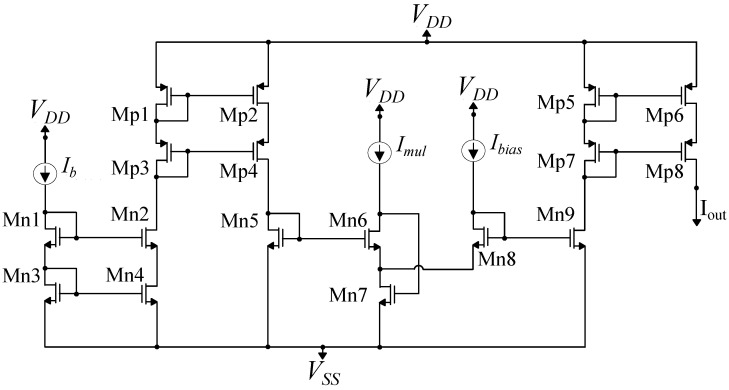
Analog multiplier circuit. To achieve accurate linear scaling, the output current of each multidimensional bump circuit is connected to this analog multiplier circuit. This implementation is based on the translinear priciple.

In order to achieve accurate linear scaling, the output current of each multidimensional (cascaded) bump circuit is connected to an analog multiplier circuit, depicted in Figure 6. The multiplier is a translinear circuit operating based on the translinear principle [39]. In particular, the translinear principle dictates that the the clockwise translinear elements’ product of the currents in a translinear loop is equal to the counterclockwise translinear elements’ product of the currents that is derived in this loop. In essence, the translinear principle in subthreshold MOS transforms the sum of gate-to-source voltages across a translinear loop into the product of currents. The sum of gate-to-source voltages across the loop is a result of Kirchhoff’s voltage law applied around the loop. Its translation to a product of currents is possible due to the exponential characteristics of the subthreshold MOS current with respect to its gate-to-source voltage.

In the proposed translinear multiplier circuit, transistors $M_{n5}$, $M_{n6}$, $M_{n8}$, and $M_{n9}$ form a translinear loop with a so-called alternating loop topology that produces an output current independent of the subthreshold slope factor κ. Furthermore, cascode NMOS and PMOS current mirrors (transistors $M_{n1}$–$M_{n4}$ and $M_{p1}$–$M_{p8}$) have been used to achieve precise current mirroring. Supposing that all four transistors ($M_{n5}$, $M_{n6}$, $M_{n8}$, and $M_{n9}$) operate in the subthreshold region and based on the translinear principle, the multiplier’s output current is the following:

(5) $$I_{out}=\frac{I_{b}I_{bias}}{I_{mul}},$$

where $I_{b}$ is the cascaded bump circuit’s output current, $I_{bias}$ is the multiplying term, and $I_{mul}$ is a normalizing current with a constant value. Transistor $M_{n7}$ is used for proper biasing of the translinear loop. The multiplier circuit’s transistor dimensions are summarized in Table 2.

In the case of GMM-based classifiers’ architectures, the peak of the RBF’s output current is controlled via the bias current of the cascaded bump architecture’s first bump cell [15,44]. Instead of this, in this work, the first bump circuit is biased with a constant bias current of 16 nA. Then, the output current of the cascaded bump is inserted as $I_{b}$ to the multiplier circuit of Figure 6, which is also biased with a constant bias current $I_{mul}$ = 16 nA. Thus, the height of the RBF cell’s output current is determined by the multiplier’s input current $I_{bias}$. This current corresponds to the Lagrange multipliers and is derived from SVM’s update rule.

**Figure 7:**
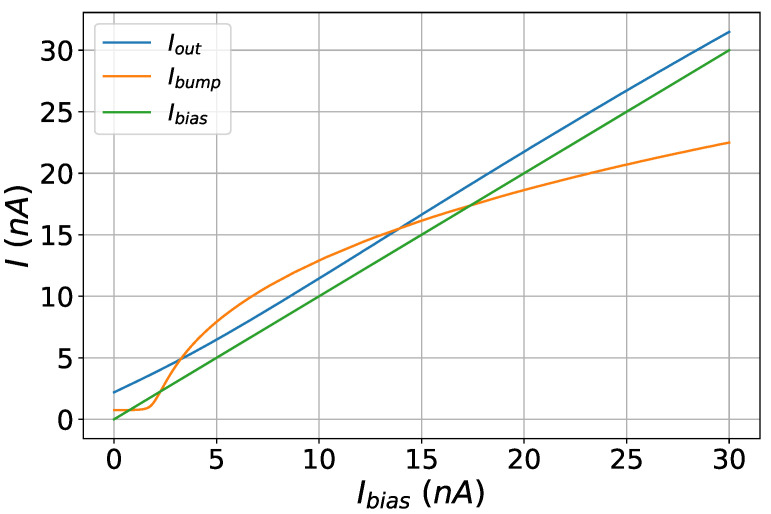
Effect of multiplier on output current of the multidimensional bump circuit. It provides a linear output current that has the same behavior with the $I_{bias}$ current.

The contribution of the multiplier circuit in achieving linear scaling of the RBF cell’s output current is evident in Figure 7. In this figure, the maximum of a 16−D RBF cell’s output current is depicted. $I_{bump}$ is the output current of the 16−D cascaded bump circuit when its peak is scaled by the bias current of the first bump circuit of the cell. $I_{out}$ is the peak of the output current if a multiplier is used. The desirable linearity is achieved, with the output current having only a small and constant dc offset compared to $I_{bias}$, which is the desired output of the multiplier.

### 4.3. Switch Cell

In the learning block, in order to satisfy the hardware-friendly SVM update rule, the product of the two learning samples’ labels has to be multiplied with each kernel. As the labels of all learning samples are either 1 or −1, the result of this product is either the positive or the negative value of the kernel that corresponds to these specific learning samples. Thus, the output current of each RBF cell that represents the kernel’s value is driven as a positive value $I_{y}$ or as a negative value $I_{x}$, depending on the aforementioned product. The positive value $I_{y}$ corresponds to $Y_{1}=Y_{2}$, while the negative value $I_{x}$ corresponds to $Y_{1}=-Y_{2}$. The labels are represented with voltages, with a positive label corresponding to the positive power supply voltage (300 mV) and a negative label corresponding to the negative one (−300 mV).

**Figure 8:**
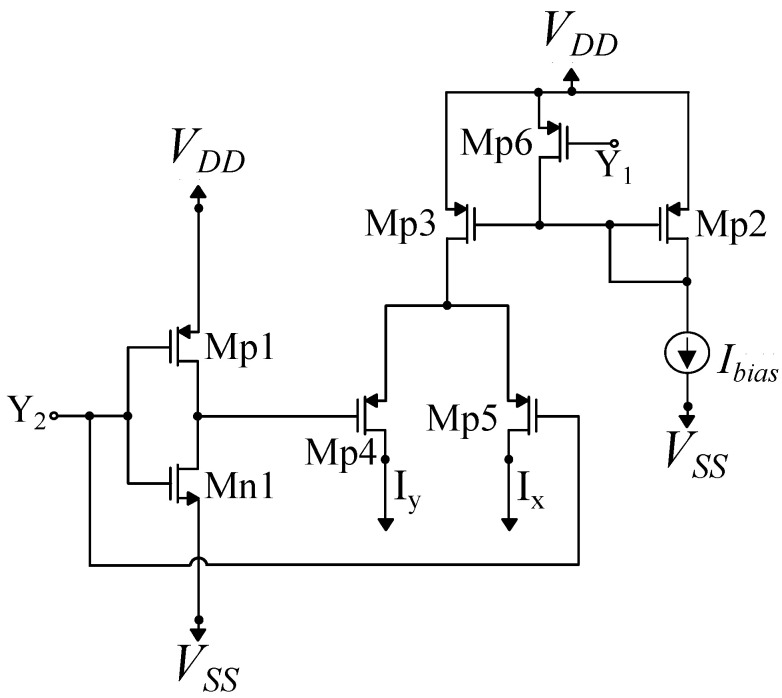
The circuit used to implement the switch cell is presented. This is a compact gate with only 6 transistors. For inputs Y1=Y2=300 mV, RBF’s current $I_{bias}$ flows through $M_{p5}$ as $I_{y}$, while for inputs Y1=−Y2=300 mV, RBF’s current $I_{bias}$ flows through $M_{p4}$ as $I_{x}$.

The selective driving of the RBF cell’s current through either $I_{y}$ or $I_{x}$ is achieved via a switch circuit [39]. The switch circuit is depicted in Figure 8 and essentially implements an compact switch. Each switch circuit receives as inputs the labels of the two learning samples of the RBF cell with which it is connected. For inputs Y1=Y2=300 mV, RBF’s current $I_{bias}$ flows through $M_{p5}$ as $I_{y}$, while for inputs Y1=−Y2=300 mV, RBF’s current $I_{bias}$ flows through $M_{p4}$ as $I_{x}$. For inputs Y1=Y2=−300 mV and Y1=−Y2=−300 mV, RBF’s current $I_{bias}$ is equal to 0 nA, since a PMOS switch is used to power-down the current mirror. This switch implementation is more compact than the one implemented with CMOS static logic, as it consists of 6 transistors instead of 8. The switch cell’s transistor dimensions are summarized in Table 3.

### 4.4. Adjuster Circuit

**Figure 9:**
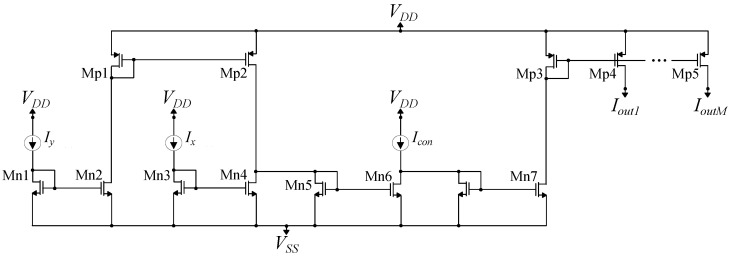
The adjuster circuit is presented. This circuit performs the non-linear minimum and maximum operations and also performs iterations based on mathematical equations, forming a feedback loop to update the current values.

**Figure 10:**
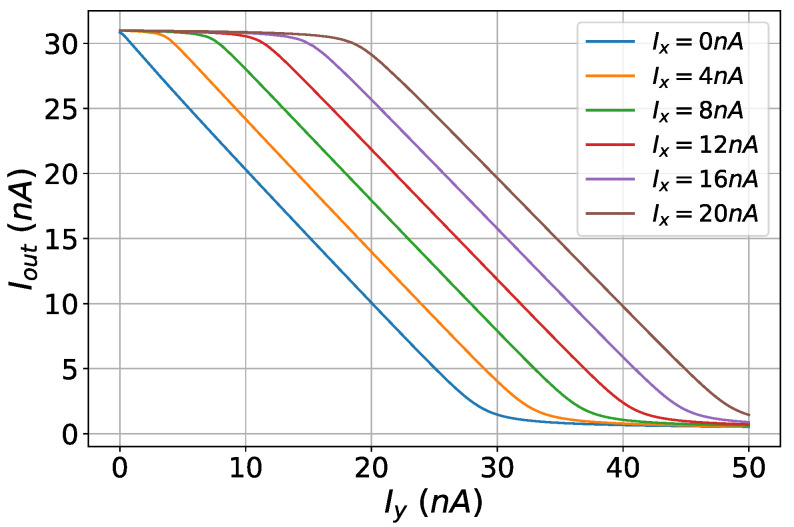
The output current of the adjuster circuit.

The hardware-friendly SVM update rule of Equation (3) can be transformed in the following current-mode equation:

(6) $$I_{new_{i}}=min\left(I_{con},max\left(0,I_{con}-y_{i}\sum_{i\neq m}y_{m}I_{m}\right)\right),$$

where $I_{new_{i}}$ is the updated value of the bias current of the $i_{th}$ RBF cell, and $I_{con}$ is a parameter current corresponding to regularization parameter *C* of the SVM. The adjuster is the circuit that performs the non-linear minimum and maximum operations as well as iterations on the above-mentioned equation, forming a feedback loop to update the current values [39]. The adjuster circuit is shown in Figure 9 and its dimensions are summarized in Table 4. It is a current mirror-based circuit with constant bias current $I_{con}$ = 40 nA and the following input currents:

(7) $$I_{y}=\sum_{y_{i}=y_{m}}I_{m},$$

(8) $$I_{x}=\sum_{y_{i}\neq y_{k}}I_{k}$$

for the *i*th adjuster circuit. The min and max operations are realized thanks to the unilateral current flow in NMOS transistors $M_{n6}$, whose current can not be lower than zero, and $M_{n7}$, whose current may not exceed the value of $I_{con}$. The proper operation of the adjuster circuit for the input current $I_{y}$ and different values of $I_{x}$ and $I_{con}$ = 30 nA is demonstrated in Figure 10. The adjuster circuit exhibits the desirable behavior based on the following expression:

(9) $$I_{out}=min(I_{con},max\left(0,I_{con}-I_{y}+I_{x}\right).$$

### 4.5. Winner-Take-All Circuit

The WTA circuit receives *N* input signals and presents in the output the response of only the largest input signal while suppressing the responses of the other N−1 inputs. In essence, the WTA circuit implements the argmax function.

There have been several voltage-mode WTA circuit implementations [40] as well as current-mode WTA circuits [45] and an ultra-low-supply voltage implementation (only 0.3 V) [46]. All such current-mode WTA circuit architectures are modifications of the original WTA circuit presented by Lazzaro [40].

**Figure 11:**
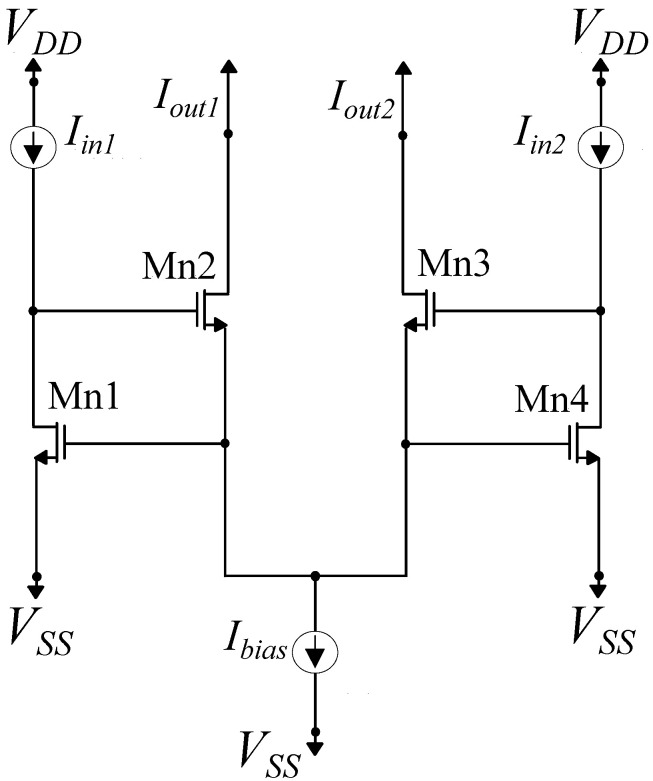
Simple NMOS winner-take-all circuit composed of two neuron cells. It is suitable for a 2-class classification problem.

**Figure 12:**
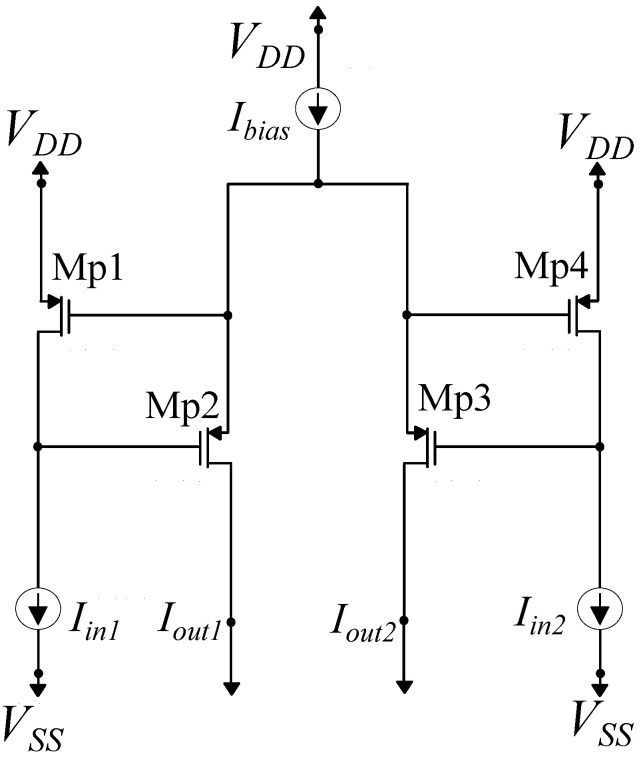
Simple PMOS winner-take-all circuit composed of two neuron cells. It is suitable for a 2-class classification problem.

The circuit architectures of the NMOS- and PMOS-based variance of the WTA circuit for two inputs are presented in Figure 11 and Figure 12, respectively. For the NMOS case, the simple WTA circuit is composed of 4 NMOS transistors of the same W and L parameters operating in the subthreshold region, and it is biased by a constant current $I_{bias}$. The transistors’ dimensions are $\left(W/L\right)=\frac{400\;\mathrm{nm}}{1600\;\mathrm{nm}}$. For equal input currents $I_{in1}=I_{in2}$, the output currents are $I_{out1}=I_{out2}=0.5I_{bias}$. Due to the fact that $M_{n1}$ and $M_{n4}$ have the same $V_{GS}$ voltage, for input currents $I_{in1}>I_{in2}$, it follows that $V_{D_{Mn1}}=V_{G_{Mn2}}>V_{G_{Mn3}}=V_{D_{Mn4}}$. Supposing that both output transistors $M_{n2}$ and $M_{n3}$ operate in saturation and, due to the fact that they both have the same source voltage, a small difference in their gate voltages results in an exponentially larger difference in the output currents. In this case, $I_{out1}=I_{bias}$ and $I_{out2}=0$. Thus, for input currents differing by a sufficient amount, only the output current corresponding to the largest input current will be non-zero.

**Figure 13:**
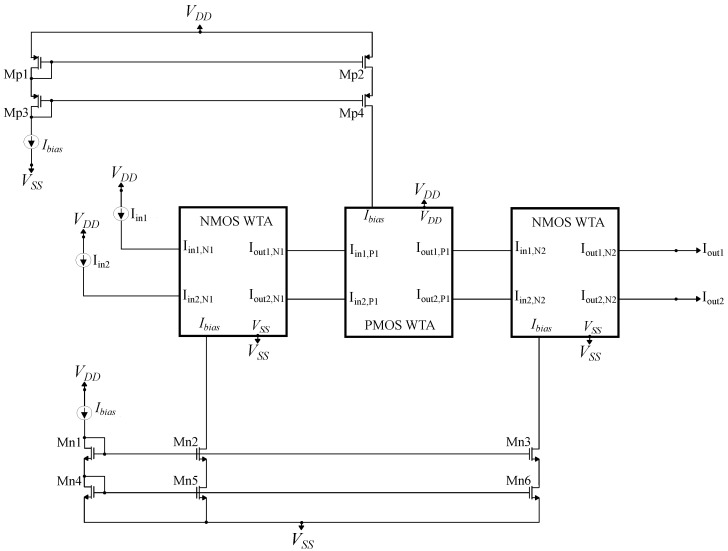
The implemented triple cascaded WTA circuit built by alternating the simple NMOS and PMOS WTA designs.

**Figure 14:**
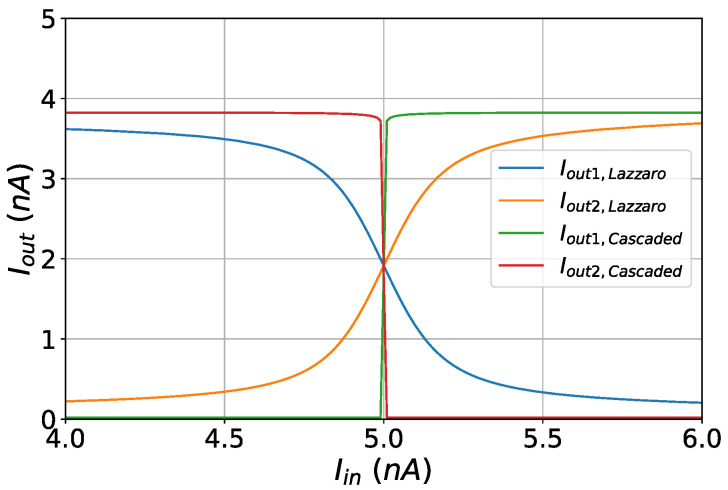
A comparison between the simple and the implemented WTA circuits.

The WTA circuit can be extended to accommodate multiple inputs. In our case, however, two inputs are required in order for the circuit to compare the positive and the negative kernel values and perform classification based on the SVM decision rule. In the proposed circuit architecture, instead of using a simple NMOS or PMOS WTA circuit, a triple WTA circuit, depicted in Figure 13, is used. It consists of an NMOS, a PMOS, and another NMOS WTA circuit connected in series, with the output currents of the one WTA block being the input currents to the next one. All 3 WTA blocks are biased with the same constant $I_{bias}=40\;\mathrm{nA}$ and essentially perform the argmax function 3 consecutive times. In Figure 14, it can be observed that by using the triple WTA circuit as opposed to the simple architecture, the minimum current difference required by the WTA system to differentiate its inputs is cut down significantly. As a result, the accuracy of the classification procedure and the quality of the digital output are increased.

## 5. Application Examples and Simulation Results

**Figure 15:**
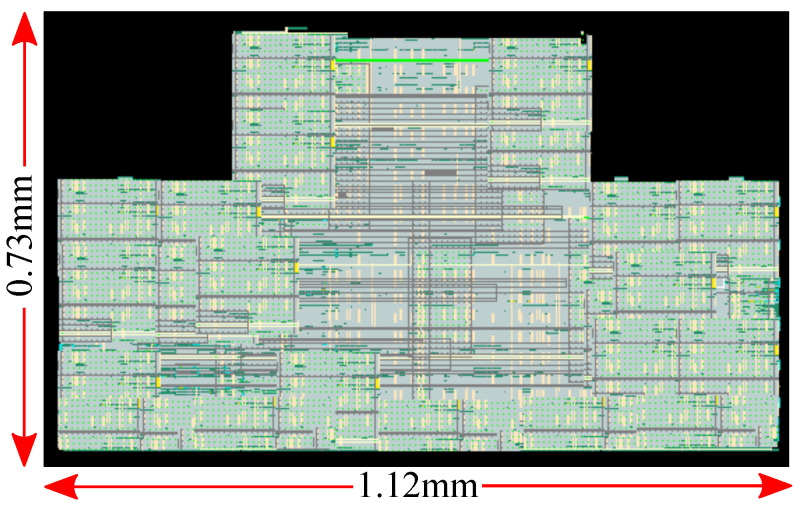
Layout of the proposed hardware-friendly SVM algorithm based on the design methodology (extra dummy transistors are used).

In this section, the proposed circuit is tested in terms of both classification accuracy and circuit sensitivity. To do so, the real-world bearing vibration data under time-varying rotational speed conditions (VSBD) [41] dataset found on the Mendeley Data website [47] is used. The dataset is composed of vibration signals measured by an accelerometer that was directly attached to the motor. These signals can be used to predict the motor’s operating condition, specifically identifying whether the motor is healthy or damaged on the inner or outer raceway. However, since the SVM algorithm’s primary usage involves binary classification problems, in this work, the motor’s condition is classified as operating correctly or faulty (with no distinction between an inner or an outer raceway defect). The layout that was used for the simulations is shown in Figure 15. Its implementation is based on the common-centroid technique, and extra dummy transistors are used in order to avoid mismatches and manufacturing considerations [48].

The data were processed before being used to train the classifier. In particular, the drive-end accelerometer data included multiple 10-s-long time series entries that are split into 10 1-second segments. The sample rate for the accelerometer was $200\times 10^{3}$ samples per second, which greatly exceeded the needs of this application and therefore were down-sampled. Finally, from each segment, the 13 features shown in Table 5 are extracted, and a random train–test slit is used to train and validate both the analog and the software-based SVMs (which will be used for comparison purposes).

**Figure 16:**
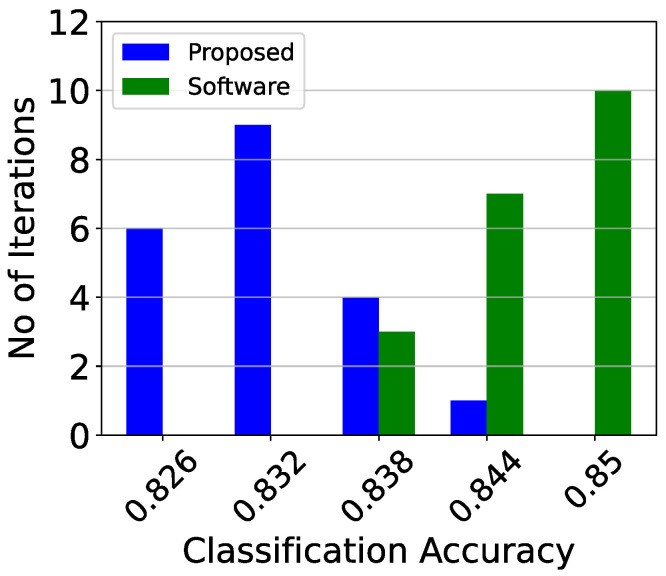
The classification results comparing the software and the proposed implementations for 20 iterations.

The analog classifier needs to be tested both as a classifier and as an analog circuit. Therefore, first, the training procedure is repeated 20 independent times to provide a robust classification accuracy and minimize random effects caused by it. In each iteration, both the analog and the software implementations are compared using the same training and validation data. Table 6 summarizes the results of this test. It is evident that the results of the hardware implementation of the proposed classifier are approximately 1% less accurate that those of an identical software-based implementation. Additionally, the deviation of their results for different train–test iterations is similar. For a more detailed comparison, the exact classification accuracy histograms are presented in Figure 16.

**Figure 17:**
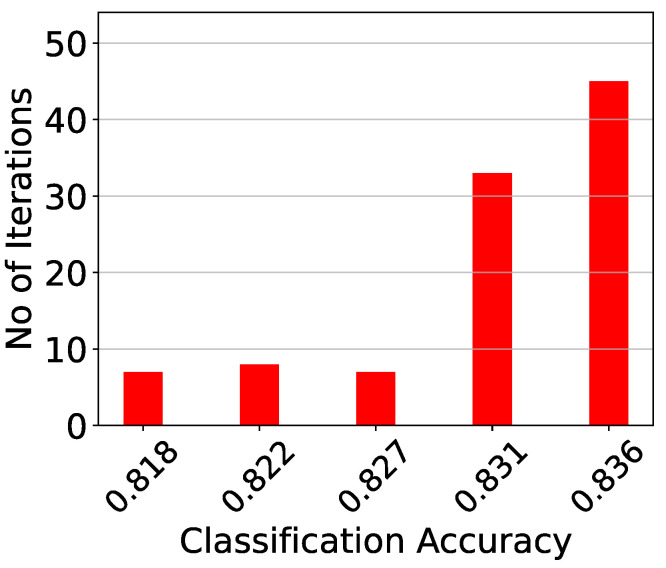
Post-layout Monte Carlo simulation results of the proposed architecture (for one of the previous 20 iterations).

A Monte Carlo analysis was conducted for the second test with N=100 points to verify the sensitivity behavior of the classifier circuit. This test used the training data of one of the 20 candidates from the previous test as input. The results are illustrated by the Monte Carlo histogram depicted in Figure 17. Its mean value is $\mu_{M}=83.2\%$, which is close to the previous test’s mean value, and the standard deviation is as low as $\sigma_{M}=0.5\%$. In general, these results demonstrate the highly sensitive behavior of the classifier. Additionally, the classifier demonstrates “systematic robustness” where, even if the internal sub-circuits are not entirely robust, as long as they behave similarly with each other, the overall classifier will output robust results. Therefore, the total system’s results are presented. In terms of corners, the worst-case scenario is slow cold, where all transistors are in the slow corner and operating at −35 °C. Here the classification accuracy is equal to 81.7%. Conversely, in the case of fast hot, where all transistors are in the fast corner and operating at 150 °C, the classification accuracy is 84.2%.

## 6. Performance Summary and Discussion

In this section, a performance summary of recent analog and mixed-mode SVM algorithms, along with that in this work, is provided. All the classifiers presented in this work are based on a hardware-friendly kernel function of the SVM algorithm. Nonetheless, it is worth mentioning that a fair comparison between hardware-based ML implementations is not possible, since there are numerous aspects that need to be considered combinatorially, such as the implemented technology, the application, the power and area specifications, the computation speed, and so forth. A performance summary for recent existing hardware-friendly SVM algorithm implementations is provided in Table 7. The aim of this work is the implementation of a power- and area-efficient classifier. As a result, subthreshold region techniques are used in order to provide a power-efficient system with minimum power supply (only 0.6 V). However, due to the complexity of the training block, the power consumption is equal to 72 μW.

The total power includes the entire classifier with biasing circuits but excludes analog memories and pre-processing circuits. In Table 7, only one classifier has a lower power consumption [31] at the cost of a larger chip area. On the other hand, the more area-efficient implementation [33] has a higher power consumption and provides a smaller number of classifications per energy unit consumed. Thus, this design provides a trade-off between high accuracy and power-area efficiency, which can be given as a summary.

The main characteristics of the classifiers presented in Table 7 are analyzed in the Introduction. Regarding the power and area of the proposed circuit, the number of support vectors and their dimensions affect these metrics. While an exact equation cannot be derived, we can predict that the power consumption and chip area are a function of $n^{2}$ with respect to the number of SVs and a function of *n* with respect to their dimensions.

The proposed training method is highly parallel, so in practice, the number of support vectors (training samples) has little effect on the training speed, which is approximately 0.3 μs. This also applies to the classification procedure. However, the number of dimensions directly affects the processing speed. Specifically, each additional dimension adds approximately 0.5 μs to the overall settling time. The proposed classifier can achieve a processing speed of 140 $K\frac{classifications}{second}$, with a settling time of approximately 7.1 μs.

## 7. Conclusions

In this work, a low-power analog integrated implementation of the SVM algorithm with on-chip learning capabilities was introduced. It utilizes the learning block, which consists of an array of RBF cells, switches, and adjuster circuits, and the classification block, which consists of RBF cells, switches and a WTA circuit. Its classification parameters were generated by on-chip training using the hardware-friendly SVM algorithm. The proposed architecture is applied to a real-world dataset targeting bearing fault diagnosis. Two main tests were conducted related to classification accuracy and sensitivity to variations and mismatches. All post-layout simulation results were extracted using the Cadence IC Suite in a TSMC 90 nm technology.

## Figures and Tables

**Table 1:** Bump circuit transistors’ dimensions.

NMOS Differential Block	W/L $(\mu_{m}/\mu_{m}$)	Current Correlator	W/L $\left(\mu_{m}/\mu_{m}\right)$
$M_{n1}$, $M_{n4}$	1.6/0.4	$M_{p3}$, $M_{p4}$	0.4/1.6
$M_{n2}$, $M_{n3}$	0.8/0.4	$M_{p1}$, $M_{p2}$	1.2/1.6
$M_{n5}$–$M_{n8}$	0.4/1.6	$M_{p5}$, $M_{p6}$	0.4/1.6
$M_{n9}$, $M_{n10}$	1.2/1.6	-	-

**Table 2:** Multiplier’s transistor dimensions.

Current Mirrors	W/L $\left(\mu_{m}/\mu_{m}\right)$	Translinear Loop	W/L $\left(\mu_{m}/\mu_{m}\right)$
$M_{n1}$–$M_{n4}$	0.4/1.6	$M_{n5}$, $M_{n9}$	0.4/1.6
$M_{p1}$–$M_{p4}$	0.4/1.6	$M_{n6}$	3.6/1.6
$M_{p5}$–$M_{p8}$	0.4/1.6	$M_{n8}$	4/1.6
$M_{n7}$	1.2/0.8	-	-

**Table 3:** Switch’s transistor dimensions.

Transistors	W/L $\left(\mu_{m}/\mu_{m}\right)$
$M_{n1}$	0.8/0.2
$M_{p1}$, $M_{p6}$	0.8/0.2
$M_{p2}$–$M_{p5}$	0.4/1.6

**Table 4:** Adjuster’s MOS transistor dimensions.

Transistors	W/L $\left(\mu_{m}/\mu_{m}\right)$
$M_{n1}$, $M_{n2}$	0.4/6.4
$M_{n3}$, $M_{n4}$	0.4/6.4
$M_{n5}$–$M_{n7}$	0.4/6.4
$M_{p1}$, $M_{p2}$	0.4/6.4
$M_{p3}$, $M_{p4}$	0.4/6.4
$M_{p5}$–$M_{p7}$	0.4/6.4

**Table 5:** Extracted features [49].

Statistic	Equation	Statistic	Equation
Root mean square	$\mathrm{RMS}=\sqrt{\frac{1}{N}\sum_{i=1}^{N}x_{i}^{2}}$	Crest factor	$\mathrm{CF}=\frac{\max(x_{i})}{\mathrm{RMS}}$
Square root of amplitude	$\mathrm{SRA}={\left(\frac{1}{N}\sum_{i=1}^{N}\sqrt{|x_{i}|}\right)}^{2}$	Impulse factor	$\mathrm{IF}=\frac{N\cdot \max(x_{i})}{\sum_{i=1}^{N}\left|x_{i}\right|}$
Kurtosis value	$\mathrm{KV}=\frac{1}{N}\sum_{i=1}^{N}{\left(\frac{x_{i}-\mu_{x}}{\sigma_{x}}\right)}^{4}$	Margin factor	$\mathrm{MF}=\frac{\max(x_{i})}{\mathrm{SRA}}$
Skewness value	$\mathrm{SV}=\frac{1}{N}\sum_{i=1}^{N}{\left(\frac{x_{i}-\mu_{x}}{\sigma_{x}}\right)}^{3}$	Frequency center	$\mathrm{FC}=\frac{1}{N}\sum_{i=1}^{N}f_{i}$
Peak-to-peak value	$\mathrm{PPV}=\max\left(x_{i}\right)-\min\left(x_{i}\right)$	Root-mean-square frequency	$\mathrm{RMSF}=\sqrt{\frac{1}{N}\sum_{i=1}^{N}f_{i}^{2}}$
Shape factor	$\mathrm{SF}=\frac{\max(x_{i})}{\mathrm{SV}}$	Root variance frequency	$\mathrm{RVF}=\sqrt{\frac{1}{N}\sum_{i=1}^{N}{\left(f_{i}-FC\right)}^{2}}$
Kurtosis factor	$\mathrm{KF}=\frac{KV}{{\mathrm{RMS}}^{4}}$	-	-

**Table 6:** Accuracy results for the VSBD dataset (over 20 iterations).

Method	Best (%)	Worst (%)	Mean (%)	Std. (%)
Software	85.3	83.8	84.6	0.4
Hardware	84.5	82.3	83.2	0.5

**Table 7:** Performance summary.

	[34]	[32]	[33]	[31]	[30]	This Work
Technology	180 nm	180 nm	Simulation	0.5 μm	0.5 μm	90 nm
Power Supply	5 V	1.8 V	N/A	4 V	5 V	0.6 V
Power Consumption	220 μW	N/A	N/A	840 nW	5.9 mW	72 μW
Area	0.06 mm${}^{2}$	0.125 mm${}^{2}$	N/A	9.0 mm${}^{2}$	9.0 mm${}^{2}$	0.818 mm${}^{2}$
Energy per Classification	252.9 pJ	N/A	N/A	21 nJ	59 nJ	576 pJ
Kernel Function	Gaussian	Gaussian	Gaussian	Quadratic	Linear	Gaussian
Operation	Learning/Classification	Learning/Classification	Learning/Classification	Classification	Classification	Learning/Classification
No. of Classes	2	2	2	24	2	2
No. of Dimensions	2	64	2	14	256	13

## Data Availability

The data used in this study are openly available in CHB-MIT Scalp EEG Database at https://data.mendeley.com/, reference number [47].
